# Zona Pellucida Dynamics Integrate Biochemical and Clinical Indicators of Embryo Competence

**DOI:** 10.3390/jcm15052038

**Published:** 2026-03-07

**Authors:** Péter Mauchart, Krisztina Gödöny, Rita Jakabfi-Csepregi, Ákos Várnagy, Endre Sulyok, József Bódis

**Affiliations:** 1 National Laboratory on Human Reproduction, University of Pécs, H-7624 Pécs, Hungary; krisztina.godony@cdoki.hu (K.G.); jakabfi-csepregi.rita@aok.pte.hu (R.J.-C.); varnagy.akos@pte.hu (Á.V.); esulyok@t-online.hu (E.S.); bodis.jozsef@pte.hu (J.B.); 2 Department of Obstetrics and Gynecology, Medical School, University of Pécs, H-7624 Pécs, Hungary; 3 Doctoral School of Health Sciences, Faculty of Health Sciences, University of Pécs, H-7621 Pécs, Hungary; 4 János Szentágothai Research Center, University of Pécs, H-7624 Pécs, Hungary; 5 Department of Laboratory Medicine, Medical School, University of Pécs, H-7624 Pécs, Hungary

**Keywords:** zona pellucida, hatching, GDF-9, follicular fluid, time-lapse, IVF, embryo competence

## Abstract

**Background/Objectives:** Dynamic remodeling of the zona pellucida (ZP) is a fundamental biochemical and structural process during human preimplantation development; however, its quantitative characterization and clinical relevance remain incompletely defined. The objective of this study was to evaluate dynamic ZP thinning as a functional marker of embryo developmental competence and to examine its relationship with follicular fluid (FF) biomarkers and clinical pregnancy. **Methods:** This prospective observational study included 47 IVF cycles performed at a single center, yielding 64 transferred blastocysts with complete time-lapse data. ZP thickness was measured from fertilization to 120 h post-fertilization using time-lapse imaging. Two quantitative parameters were derived: the relative thinning ratio (Δrel) and the linear thinning rate (slope). FF concentrations of growth differentiation factor 9 (GDF-9), hyaluronic acid (HA), and syndecan-4 (Syn4) were quantified by ELISA. Embryo-level associations with spontaneous blastocyst hatching were assessed using logistic regression and multivariate analyses, while patient-level models evaluated predictors of clinical pregnancy. **Results:** Embryos that underwent spontaneous hatching exhibited significantly greater Δrel than non-hatching embryos (*p* < 0.001). Δrel remained the strongest predictor of hatching in multivariable models (AUC = 0.91). Among FF biomarkers, only GDF-9 showed a positive association with spontaneous hatching. At the patient level, higher Δrel values of transferred embryos were associated with clinical pregnancy (OR 3.65, *p* = 0.009), whereas FF biomarkers and assisted hatching showed no significant association. **Conclusions:** Dynamic ZP thinning quantified by Δrel represents a promising indicator of embryo developmental competence. The concordance between embryo-level hatching behavior and patient-level clinical pregnancy suggests potential clinical relevance of ZP dynamics as an integrative embryological marker, warranting validation in larger cohorts.

## 1. Introduction

Blastocyst hatching is a complex biological process that refers to the release of the developing embryo from the zona pellucida (ZP) coat to implant and to establish cell contact between trophoectoderm and endometrial epithelium [1]. The thinning and breaking of the ZP are achieved by two major mechanisms: (1) the mechanical pressure of the expanding blastocyst on the ZP and (2) chemical dissolution of the zona material by protease enzymes [2,3,4,5].

ZP is an acellular, dynamic structure consisting of interconnected fibrils and glycoproteins. It has an essential role in folliculogenesis, fertilization and preimplantation development. It mediates capacitated sperm binding to the ovulated oocyte, blocks post-fertilization polyspermy, and protects the early embryo prior to implantation [6,7,8]. In humans, four ZP glycoproteins (ZPGs) have been identified (ZP 1-4) [9,10]. ZP gene sequence variations [11,12], dysmorphology [13] and the presence of anti-ZP antibodies [14,15,16,17] have been documented to be associated with fertilization failure.

ZP thickness varies with the oocyte’s diameter and reaches 10–30 µm at fertilization [18]. The developmental pattern of ZPG synthesis during oogenesis has been defined in mice by measuring ZP3 mRNA. It has been clearly shown that the number of copies of ZPG3 mRNA per oocyte/egg steadily increased from undetectable levels in non-growing oocytes to reach its maximum in fully grown oocytes. Simultaneously with this process, continuous thickening of ZP occurred. During conversion of oocytes to unfertilized or fertilized eggs, the number of ZPG3 mRNA copies fell dramatically, indicating that the synthesis of ZPGs ceased [19].

Prior to blastocyst hatching, natural thinning of ZP is encountered that may help the early embryo to leave the ZP envelope. With this notion in line, associations have been published between the thickness of ZP and the rate of successful implantation and pregnancy. However, important studies argue against this possibility and provide convincing evidence that the IVF outcome is independent of the ZP thickness. The conflicting results can be reconciled by the opposing effects of pre-hatching hardening of ZP that have been shown to be intensified by IVF culture media and the freezing-rewarming of the embryos [20,21,22,23,24,25,26].

To obtain additional information on the relationship between ZP thickness and blastocyst hatching/implantation the present study was designed to determine: (1) the linear thinning rate and the relative thinning ratio of ZP, (2) the possible contribution of oocyte-specific growth and differentiation factor (GDF-9), and (3) the levels of essential components of ZP in follicular fluid (syndecan-4, hyaluronic acid) that may derive from the normal ZP metabolism or shed by disintegrated ZP.

The primary objective of this study was to evaluate dynamic zona pellucida thinning as a quantitative marker of embryo competence, with particular emphasis on its association with spontaneous blastocyst hatching. A secondary objective was to assess whether embryo-level zona pellucida dynamics and related follicular fluid biomarkers are consistent with patient-level clinical pregnancy outcomes.

## 2. Materials and Methods

### 2.1. Study Design, Setting, and Participants

This was a prospective observational analysis of 47 IVF cycles performed in 2022 at a single IVF center. All cycles had day 5 embryo transfers and complete embryology records. Inclusion criteria were: (1) availability of time-lapse imaging from fertilization to 120 h; (2) paired serum and follicular fluid (FF) samples collected at oocyte pick-up (OPU); and (3) documented stimulation parameters and outcome. One or two embryos were transferred per cycle according to routine clinical criteria.

No patients were excluded based on age, ovarian reserve parameters, baseline hormonal status, or expected clinical prognosis. This approach was deliberately chosen to capture the full biological variability of zona pellucida remodeling across different clinical contexts, rather than applying prognostic preselection.

Demographic and clinical variables recorded at the patient-level included maternal age, BMI, stimulation protocol (antagonist, agonist short, agonist long), and cumulative FSH dose (IU) during the stimulation cycle. Serum estradiol (E2) was measured on days 6–8 of the stimulated cycle and entered into models as ‘mid-stimulation E2’. Additional recorded variables were the number of retrieved oocytes, the number of mature (MII) oocytes, and whether cryopreservation was performed. Of the 47 cycles included in the study, 12 were performed using conventional IVF, 33 using ICSI, and 2 involved a mixed fertilization approach.

A total of 90 blastocysts were obtained during the study period; however, only transferred blastocysts (*n* = 64) were included in the present analyses.

### 2.2. Controlled Ovarian Hyperstimulation

Before starting the stimulation, all patients underwent routine assessments, including cervical cytology, serological screening for HIV and hepatitis B, uterine cavity assessment by hysteroscopy or HyCoSy, and male factor evaluation. Baseline hormonal evaluation was performed as part of routine clinical assessment and was used to guide standard IVF management. Controlled ovarian stimulation was carried out with GnRH agonist triptorelin in the course of both long and short protocols, and GnRH antagonist protocol using cetrorelix. Follicular development was induced with individualized daily doses of recombinant FSH (rFSH, typically 150–250 IU), adjusted according to BMI and age; in patients with a history of poor ovarian response, the dose could be increased up to 300 IU. Recombinant LH or hMG was added if clinically indicated. From stimulation day 6, follicular growth was monitored by ultrasound every second day. When at least two follicles reached ≥17 mm in diameter, final oocyte maturation was triggered with 250 µg (≈6500 IU) recombinant hCG. Oocyte retrieval followed 36 h later, performed under sedation by transvaginal ultrasound-guided aspiration.

### 2.3. Embryological Procedures, Embryo Transfer and Clinical Outcomes

Oocytes were collected in G-MOPS handling medium, and all procedures were performed using Vitrolife “G” series culture media (Göteborg, Sweden). Fertilization was achieved either by conventional IVF or by intracytoplasmic sperm injection (ICSI), depending on sperm quality parameters (e.g., low concentration, motility, or elevated DNA fragmentation index), maternal age (>35 years), or a history of repeated IVF failure (>2 cycles). For ICSI, only mature (MII) oocytes showing the first polar body were injected following cumulus removal with hyaluronidase. Fertilization was performed 2–3 h after oocyte retrieval. Conventional IVF was carried out in bicarbonate-buffered G-IVF medium, and successful fertilization was verified the following day by the presence of two pronuclei (2PN) in GTL medium. The fertilization rate was expressed as the proportion of normally fertilized (2PN) oocytes relative to the number of injected or inseminated oocytes. Embryos were cultured in single-step GTL medium (Vitrolife, Göteborg, Sweden) under reduced oxygen tension (5% O_2_, 6% CO_2_, 89% N_2_) in an EmbryoScope time-lapse incubator. The system provided continuous culture in individually allocated microwells within a temperature-controlled environment (37 °C). Time-lapse imaging was performed automatically at predefined intervals (every 10 min) using a multi-focal acquisition scheme (11 focal planes), without disturbing the embryos, allowing uninterrupted monitoring of developmental kinetics and zona pellucida dynamics. Embryo transfer was carried out exclusively on day 5 at the blastocyst stage. According to patient preference, a maximum of two blastocysts were transferred. Assisted hatching (AH) was not performed uniformly for all embryos; therefore, an ‘AH’ variable was coded based on the clinic’s policy (maternal age ≥37 years, ≥3 previous IVF failures, or ZP thickness >17 μm at 120 h). This variable was used as a covariate in the pregnancy-level regression model. No AH procedure was performed on embryos outside these indications. Luteal support consisted of daily vaginal progesterone supplementation (400–800 mg). Clinical pregnancy was assessed as described in Section 2.6.

### 2.4. Sample Collection and Biomarker Assays

At oocyte retrieval, matched serum and FF were collected, clarified by centrifugation, aliquoted, and stored at −80 °C until batch analysis. Follicular fluid (FF) biomarkers (GDF-9, HA, Syn4) were measured at the cycle level. Accordingly, all embryos originating from the same cycle were assigned identical FF biomarker values in embryo-level analyses. Concentrations of syndecan-4 (Syn4), growth differentiation factor-9 (GDF-9), and hyaluronic acid (HA) were determined by commercially available human enzyme-linked immunosorbent assay (ELISA) kits from Abbexa Ltd. (Cambridge, UK) in the Szentágothai Research Center (University of Pécs) following the manufacturer’s protocol. The ELISA plates were evaluated at 450 nm in a Biotek Synergy HT reader (Agilent BioTek, Santa Clara, CA, USA).

### 2.5. Time-Lapse Imaging and ZP Measurements

Zona pellucida (ZP) thickness was quantified from EmbryoScope image exports at six standardized post-fertilization time points (0, 24, 48, 72, 96, and 120 h). At each time point, three measurements were taken and averaged to obtain a single representative value (“avg” columns in the raw dataset). From these values, we derived two quantitative indices per embryo:

#### 2.5.1. Relative Thinning Ratio (Δrel)

Relative thinning ratio (Δrel): Δrel = (ZP0h − ZP120 h)/ZP0 h

where ZP0h is the zona thickness (μm) at fertilization, and ZP120 h is the thickness at 120 h.

#### 2.5.2. Linear Thinning Rate (Slope)

This parameter represents the rate of thinning over time. Conceptually, it is defined as the change in thickness (ΔZP) over the temporal interval (Δt, in hours). In practice, the slope is given by the regression coefficient of a linear regression model, where t denotes time (hours) and y the measured ZP thickness:

ZP(t) = β0 + β1 t + ε, t ∈ {0, 24, 48, 72, 96, 120}

where the coefficient β1 (μm/h) reflects the average rate of thinning (more negative = faster thinning), and ε represents the residual error term of the regression model. Linear fits were chosen for interpretability and robustness across the six averaged time points.

### 2.6. Outcomes

The primary clinical outcome was patient-level pregnancy, defined as a positive serum β-hCG test 11–14 days after embryo transfer and confirmation of an intrauterine gestational sac on ultrasound three weeks later. At the embryo level, spontaneous hatching (yes/no) was recorded based on time-lapse observation of blastocyst emergence from the zona pellucida. Logistic regression models evaluating pregnancy were therefore fitted at the patient level, whereas models of hatching were fitted at the embryo level.

### 2.7. Statistical Analysis

#### 2.7.1. Data Handling and Simple Statistical Tests

Continuous variables were summarized as mean ± standard deviation (SD) or median with interquartile range (IQR), depending on distribution. Group comparisons (e.g., hatching vs. non-hatching; pregnant vs. non-pregnant) were performed using Welch’s *t*-tests, allowing for unequal variances. Continuous predictors were z-standardized prior to ordination or regression modeling, and skewed biomarkers were log-transformed when entered into analyses.

#### 2.7.2. Logistic Regression Models

At the embryo level, logistic regression was used to evaluate whether zona pellucida dynamics predicted spontaneous hatching. Standardized Δrel was used as the primary predictor, reflecting zona thinning as the central descriptor of the process, and follicular fluid biomarkers (GDF-9, Syn4, HA) were additionally considered as covariates (entered on the log scale where appropriate). Embryo-level analyses were restricted to transferred blastocysts. Because up to two embryos originated from the same patient, potential non-independence was addressed by a one-embryo-per-patient sensitivity analysis for the spontaneous hatching outcome, in which a single embryo was randomly selected per patient, and the model was refit.

At the patient level, logistic regression was used to assess whether ZP dynamics and FF biomarkers predicted clinical pregnancy. The dependent variable was clinical pregnancy (yes/no). In single embryo transfer (SET) cycles, the Δrel value of the transferred embryo was used. In double embryo transfer (DET) cycles, the primary analysis used the mean Δrel of transferred embryos to represent the overall zona pellucida thinning characteristic of the transfer cohort. As a sensitivity analysis, patient-level models were repeated using the maximum Δrel value among transferred embryos instead of the mean.

To account for within-patient dependence when multiple embryos contributed to a transfer, sensitivity analyses of clinical pregnancy models were performed using (i) generalized estimating equations (GEEs) with an exchangeable correlation structure and patient identifier as the clustering variable, and (ii) a one-embryo-per-patient approach in which a single embryo was randomly selected per patient.

Because Δrel and slope are mathematically related descriptors of the same ZP thinning process, they exhibited strong collinearity. To avoid redundancy, Δrel (standardized) was used as the primary predictor in logistic regression models, whereas slope was evaluated in supplementary analyses (e.g., PCA and GAM).

#### 2.7.3. PCA and Non-Linear Effects

To visualize the multivariate structure at the embryo-level, we performed principal component analysis (PCA) on standardized variables, including Δrel, slope, FF biomarkers (GDF-9, Syn4, HA), and selected clinical parameters (BMI, maternal age, cumulative FSH dose). PCA scores were plotted in two-dimensional space, with convex hulls delineating outcome groups. For spontaneous hatching, embryos were separated into hatching vs. non-hatching groups, whereas for clinical outcome, the same analysis was repeated with convex hulls drawn according to pregnancy status (pregnant vs. non-pregnant). To evaluate potential non-linear relationships between the PCA axes and outcomes, we fitted generalized additive models (GAMs) using smooth functions of PC1 and PC2 to predict the probability of hatching or pregnancy. Probability contours in the PC1–PC2 plane consistently showed that PC1, dominated by Δrel and slope loadings, was the principal driver of outcome discrimination, while PC2 contributed little additional information.

#### 2.7.4. CCA and Permutation Testing

We used canonical correspondence analysis (CCA) as a constrained ordination method to jointly visualize zona pellucida (ZP) dynamics and their clinical/biochemical correlates in a low-dimensional canonical space. CCA was applied to investigate how clinical and follicular fluid (FF) variables were associated with ZP remodeling. The response matrix comprised two descriptors of ZP dynamics: relative thinning (Δrel) and the slope of thinning over 0–120 h. Explanatory variables included maternal age, BMI, cumulative FSH dose, and FF concentrations of GDF-9, Syn4, and HA; stimulation protocol was entered as categorical dummy variables. Continuous predictors were z-standardized. Models were fitted on complete cases, and significance was assessed with 999 permutation tests.

At the patient level, analyses were repeated using the mean Δrel and slope values across transferred embryos, with the predictor set expanded to include the number of MII oocytes and mid-stimulation serum estradiol. For CCA models, global model fit is reported as the proportion of constrained inertia relative to total inertia (pseudo-R^2^). At the patient level, adjusted R^2^ values were additionally calculated using vegan::RsquareAdj to account for sample size and model complexity. Clinical outcomes (pregnancy, hatching) were not included as predictors but were overlaid graphically for interpretative purposes.

#### 2.7.5. Analyses for Clinical Interpretability

To enhance clinical interpretability, additional analyses were performed at the patient level. Odds ratios were estimated per 0.1 increment of Δrel using unstandardized models. Finally, Δrel was categorized into quartiles to examine pregnancy rates and test for linear trend across increasing levels of zona thinning.

All statistical calculations were performed in R 4.4.0 [27], using ggplot2, mgcv, glmnet, vegan, FactoMineR, factoextra, and pROC.

## 3. Results

The results are presented in two parts. First, embryo-level analyses examine the relationship between zona pellucida thinning dynamics and spontaneous blastocyst hatching. Second, patient-level analyses evaluate whether zona pellucida dynamics of transferred embryos and follicular fluid biomarkers are associated with clinical pregnancy.

### 3.1. Zona Pellucida Dynamics and Spontaneous Hatching (Embryo-Level)

Embryos that underwent spontaneous hatching exhibited substantially greater ZP thinning (Table 1). The mean Δrel was higher than in non-hatching embryos, and the difference was statistically significant. In parallel, the average thinning rate captured by the ZP slope was also significantly steeper in hatching embryos, further supporting the robustness of the association. Follicular fluid GDF-9 concentrations were higher in hatching embryos (≈2219 vs. 1472 pg/mL, *p* = 0.0161), whereas HA showed only a trend, and Syn4 did not differ.

#### 3.1.1. Logistic Regression and Model Performance

In logistic regression with standardized predictors, both Δrel and FF GDF-9 emerged as independent predictors of spontaneous hatching (Table 2). Δrel showed the strongest effect, while GDF-9 contributed an additional significant association. Syn4 and HA were not significant. The combined model achieved high discrimination, with an apparent AUC of 0.91 in ROC analysis, which should be interpreted as exploratory given the cohort size. To account for potential non-independence of embryos originating from the same patient, a one-embryo-per-patient sensitivity analysis was performed. Effect directions were consistent with the main analysis (Supplementary Table S1).

#### 3.1.2. PCA and Non-Linear Structure

**Figure 1:**
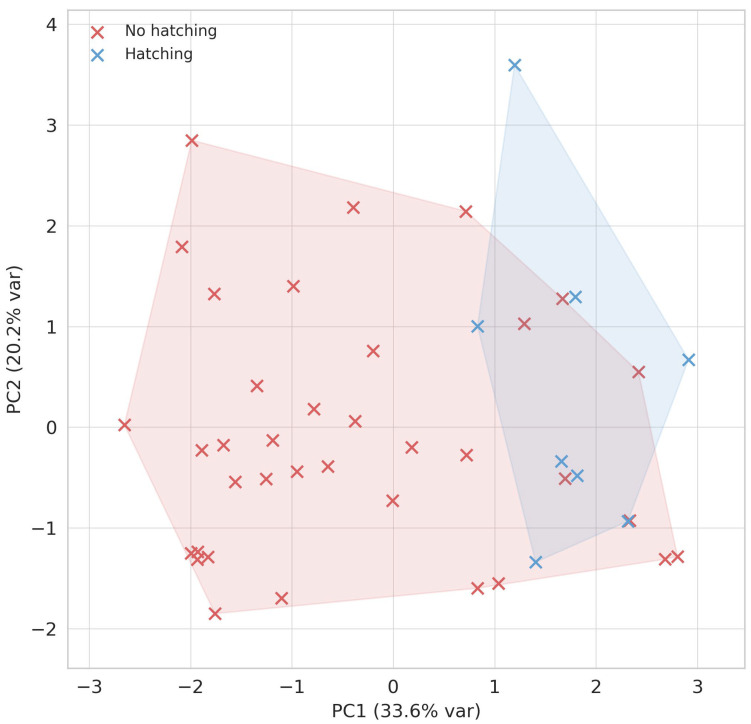
Principal component analysis (PCA) of embryo-level variables (Δrel, slope, clinical and FF predictors). Points represent embryos, with convex hulls delineating hatching vs. non-hatching groups. Axes show the first two principal components with the percentage of variance explained. Embryos were projected onto the first two principal components (PC1 explaining 33.6% of variance, PC2 20.2%). Convex hulls illustrate group separation between hatching (blue) and non-hatching (red) embryos.

PCA of embryo-level variables showed that the first two components together explained 53.8% of the variance (PC1: 33.6%, PC2: 20.2%). When embryos were grouped by outcome, convex hulls revealed a partial but distinct segregation between hatching and non-hatching embryos along PC1 (Figure 1). Hatching embryos clustered toward higher PC1 values, consistent with the interpretation of PC1 as a “ZP-dynamics axis,” whereas non-hatching embryos were shifted toward lower PC1 values with greater overlap on PC2.

The contribution of individual variables confirmed this interpretation: PC1 was dominated by zona pellucida dynamics, with the strongest loadings from Δrel (30.8%) and slope (29.3%), together with FSH dose (19.0%) and GDF-9 (8.4%), reflecting embryo-intrinsic remodeling and oocyte competence. PC2 was driven mainly by BMI (41.5%) and Syn4 (41.6%), with smaller contributions from FSH dose (8.5%), representing clinical and extracellular matrix variance (Table 3).

Generalized additive models (GAMs) fitted in the PCA space confirmed that hatching probability increased steeply and non-linearly along PC1, while PC2 showed only a marginal, non-significant trend. Probability contours in the PC1–PC2 plane matched this pattern, underscoring that embryo-intrinsic zona remodeling was the dominant determinant of spontaneous hatching (Table 4).

#### 3.1.3. CCA and Permutation Testing at Embryo-Level

**Figure 2:**
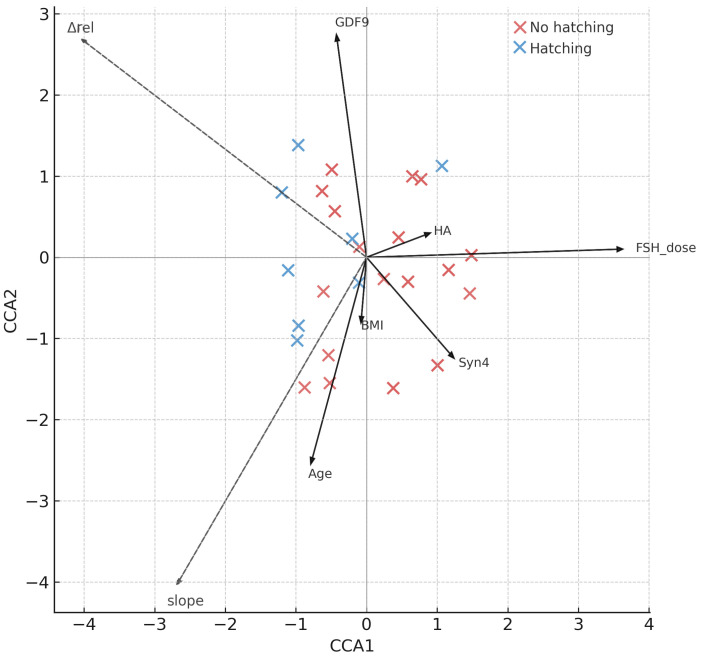
Canonical correspondence analysis (CCA) triplot of embryo-level ZP dynamics (Δrel, slope) constrained by age, BMI, FSH dose, FF GDF-9, Syn4, and HA. Arrows indicate the direction and strength of covariates; response vectors show the ZP-dynamics axis. Note: Δrel and slope represent the same thinning process but with opposite numerical orientation (higher Δrel corresponds to a more negative slope), which explains their opposing directions in the ordination space.

Canonical correspondence analysis (CCA) at the embryo-level demonstrated that clinical and biochemical predictors explained a significant proportion of the variance in zona pellucida dynamics. The global model explained 30.8% of total inertia (pseudo-R^2^ = 0.308, permutation *p* = 0.022). As illustrated in Figure 2, the main driver was stimulation intensity, with the FSH dose vector aligning strongly with the Δrel/slope axis. Follicular fluid GDF-9 also contributed, pointing in a direction consistent with greater ZP thinning, although its effect was weaker. Age showed a minor opposing influence, while BMI, HA, and Syn4 clustered near the origin, indicating little independent explanatory power. Outcome groups (hatching vs. non-hatching) were partially separated in canonical space, aligning with the Δrel and slope response vectors. These patterns highlight that stimulation dose showed the strongest association with the canonical ZP axis, with a possible additional role for oocyte–granulosa competence as reflected by GDF-9.

Permutation ANOVA of the embryo-level CCA, with ZP dynamics (Δrel and slope) as the response matrix constrained by clinical and FF predictors, confirmed a significant global model fit (*p* = 0.022; Table 5). Among the individual predictors, FSH dose showed the highest marginal contribution (*p* = 0.003), followed by follicular-fluid GDF-9 (*p* = 0.036). Maternal age showed a borderline association (*p* ≈ 0.073), while BMI, HA, and Syn4 were not significant.

### 3.2. Zona Pellucida Dynamics and Clinical Pregnancy (Patient-Level)

In contrast to hatching, clinical pregnancy was less strongly associated with FF biomarkers. GDF-9, HA, and Syn4 did not differ significantly between pregnant and non-pregnant patients (Table 1). By contrast, ZP dynamics were again informative: embryos from patients who achieved pregnancy displayed significantly higher Δrel values (0.61 ± 0.33 vs. 0.34 ± 0.30, *p* = 0.0219). The ZP slope also differed significantly between groups (−0.08 ± 0.04 vs. −0.03 ± 0.03, *p* = 0.0025), showing an even stronger statistical association than Δrel. Together, these findings indicate that both descriptors of zona pellucida remodeling consistently align with successful outcomes.

#### 3.2.1. Logistic Regression Analysis of Clinical Pregnancy

At the patient level, clinical pregnancy was associated with more pronounced zona pellucida thinning. Patients who achieved pregnancy had transferred embryos with higher mean Δrel values than non-pregnant patients (0.61 ± 0.33 vs. 0.34 ± 0.30, *p* = 0.0219; Table 1). In multivariable logistic regression, including standardized Δrel, FF GDF-9, Syn4, HA, and assisted hatching as covariates, Δrel remained the only significant predictor of clinical pregnancy (OR 3.65, 95% CI 1.39–9.62, *p* = 0.009; Table 6). None of the FF biomarkers nor assisted hatching reached statistical significance (Table 6). The model showed moderate discrimination (AUC ≈ 0.73), indicating that patients with embryos exhibiting greater ZP thinning were more likely to conceive, even after accounting for FF markers and assisted hatching. In generalized estimating equation (GEE) models accounting for patient-level clustering (Supplementary Table S2), Δrel remained significantly associated with clinical pregnancy (OR 3.72, CI 1.45–9.51, *p* = 0.006). The sensitivity analysis comparing mean vs. maximum Δrel aggregation yielded consistent results (Supplementary Table S3), indicating that the observed association was not dependent on the aggregation strategy applied in double embryo transfer cycles. Assisted hatching was indication-driven and applied predominantly in patients with advanced maternal age, repeated IVF failure, or increased zona thickness. Baseline comparisons confirmed that patients undergoing assisted hatching had significantly higher maternal age (*p* = 0.003), more previous IVF attempts (*p* < 0.001), and thicker zona pellucida at 120 h (*p* = 0.008) (Supplementary Table S4). Clinical pregnancy rates did not differ significantly between groups (*p* = 0.30). In addition to standardized models, effect sizes were examined on the original Δrel scale to enhance clinical interpretability.

In adjusted logistic regression using unstandardized Δrel, each 0.1 increase in mean Δrel was associated with a 45% increase in the odds of clinical pregnancy (OR 1.45, CI 1.10–1.91, *p* = 0.009, Supplementary Table S5A).

When Δrel was categorized into quartiles, empirical pregnancy rates varied across groups (8.3%, 33.3%, 8.3%, and 72.7% in quartiles 1–4, respectively), with the highest rate observed in the upper quartile. Model-based predicted probabilities demonstrated a consistent monotonic increase (8.7%, 18.9%, 36.1%, and 57.8%). A significant linear trend across quartiles was observed (*p* = 0.011), supporting an overall dose–response relationship between zona pellucida thinning and pregnancy likelihood (Supplementary Table S5B).

#### 3.2.2. PCA of Clinical Pregnancy Outcomes

**Figure 3:**
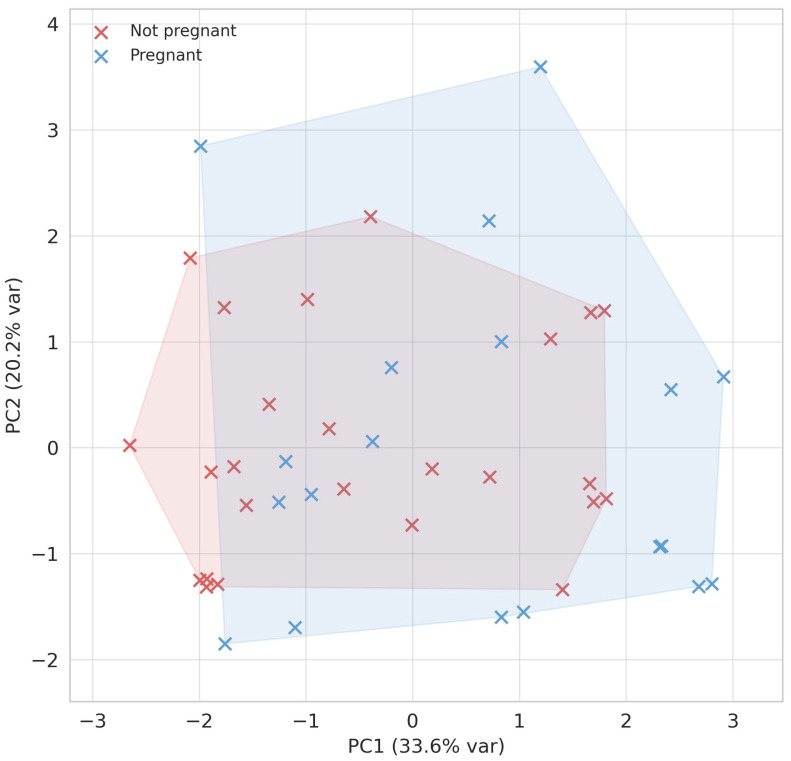
Principal component analysis (PCA) of embryo-level variables (Δrel, slope, clinical and FF predictors). Points represent embryos, with convex hulls delineating pregnant (blue) vs. non-pregnant (red) groups. Axes indicate the first two principal components and the percentage of variance explained.

The same PCA space as in the hatching analysis was used, but embryos were now grouped by pregnancy outcome (pregnant vs. non-pregnant). This revealed only modest separation between groups (Figure 3). The distinction was nevertheless discernible, with embryos from pregnant patients tending to lie toward the positive side of PC1, consistent with higher ZP-thinning dynamics. No clear differentiation was observed along PC2.

#### 3.2.3. CCA and Clinical Covariates at Patient-Level

**Figure 4:**
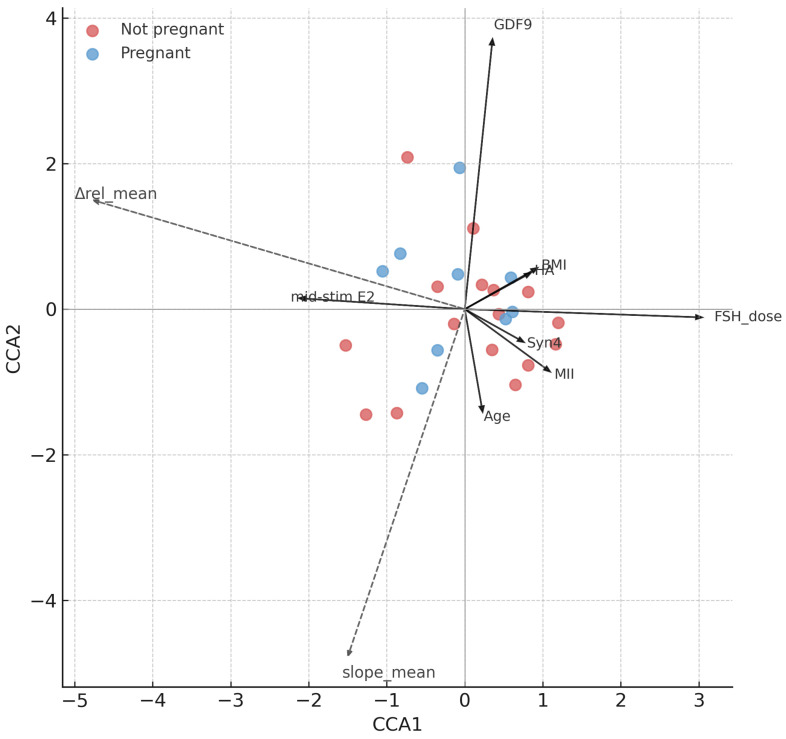
Canonical correspondence analysis (CCA) triplot of patient-level ZP dynamics. Response variables were Δrel_mean and slope_mean; explanatory variables included maternal age, BMI, FSH dose, follicular fluid biomarkers (GDF-9, Syn4, HA), number of MII oocytes, mid-stimulation estradiol, and stimulation protocol. Arrows represent the direction and relative contribution of predictors; points represent patients, colored by pregnancy outcome (pregnant vs. not pregnant). Note: Δrel and slope represent the same thinning process but with opposite numerical orientation (higher Δrel corresponds to a more negative slope), which explains their opposing directions in the ordination space.

At the patient-level, canonical correspondence analysis (CCA) indicated that clinical and biochemical predictors explained a substantial proportion of the variance in ZP dynamics (adjusted R^2^ = 0.28, *p* = 0.005). As shown in Figure 4, the FSH dose vector aligned most strongly with the Δrel_mean and slope_mean response variables, confirming stimulation intensity as the dominant driver. Maternal age also contributed independently, projecting into the same canonical space but with a weaker effect. Follicular fluid GDF-9 pointed toward greater ZP remodeling, consistent with a potential supportive role, although this association was only borderline significant. Mid-stimulation estradiol showed a minor contribution, whereas BMI, MII count, HA, Syn4, and protocol type clustered near the origin, indicating little explanatory value. Pregnant vs. non-pregnant patients showed partial separation along the canonical axes, with pregnant cases tending toward higher Δrel_mean and steeper slope_mean, aligning with the directions of FSH dose and GDF-9. These findings suggest that patient-level ZP dynamics are primarily shaped by stimulation intensity and maternal age, with a possible contribution from FF GDF-9, while other clinical or biochemical variables appear less influential.

At the patient level, the constrained ordination model explained 28% of the variance in ZP dynamics (adjusted R^2^ = 0.28), and the global model was significant by permutation ANOVA (*p* = 0.005; Figure 4; Table 7).

Among individual predictors, cumulative FSH dose was the dominant clinical factor (*p* = 0.004), followed by maternal age (*p* = 0.045). Follicular fluid GDF-9 showed a borderline association (*p* = 0.087), whereas BMI, MII oocyte number, hyaluronic acid, Syn4, mid-stimulation estradiol, and stimulation protocol were not significant.

These findings suggest that stimulation intensity and maternal age are the primary clinical determinants of patient-level zona pellucida dynamics, with a possible minor contribution from GDF-9.

## 4. Discussion

The human zona pellucida is a specialized extracellular matrix primarily composed of glycoproteins ZP1–ZP4, whose structural and biochemical remodeling underlies the dynamic changes in zona thickness observed during preimplantation development [9]. The present study provided convincing evidence that the parameters of ZP dynamics, the linear thinning rate, and the relative thinning ratio of ZP have better predictive power for spontaneous blastocyst hatching than the ZP thickness alone. Furthermore, follicular fluid GDF-9 levels were associated with spontaneous hatching, whereas no independent association was observed with clinical pregnancy. It is to be noted, however, that the predictive value of ZP dynamics and FF GDF-9 for pregnancy is not as robust as it is for hatching.

As shown in Table 1, embryos with spontaneous hatching displayed consistently greater relative zona pellucida thinning (Δrel) than non-hatching embryos, whereas differences in other examined parameters were less pronounced. This finding highlights Δrel as the most informative single descriptor of zona pellucida remodeling in the present study.

In our patient-level multivariable model, assisted hatching was not independently associated with clinical pregnancy. However, assisted hatching in our cohort was indication-driven and predominantly applied in patients with advanced maternal age, repeated IVF failure, or increased zona thickness. Baseline comparisons confirmed significant differences between AH and non-AH groups (Supplementary Table S4). Therefore, the absence of a significant association should not be interpreted as evidence of a lack of efficacy but rather as reflecting confounding by indication.

These observations may have clinical relevance, particularly in conditions where assisted hatching is considered, but with an uncertain effect on implantation and pregnancy outcome. These conditions include cycles with poor embryo quality [28,29,30], advanced maternal age (>38 years) [31,32,33], diminished ovarian reserve [34,35], and recurrent IVF failures [29,36,37,38]. However, given the observational design of the present study, causal conclusions regarding the effectiveness of assisted hatching cannot be drawn.

In our study, attempts have also been made to reveal the possible involvement of GDF-9 in embryo hatching and in subsequent early embryo development. GDF-9 forms a biologically active heterodimer with bone morphogenetic protein-15 (BMP-15), and they interact synergistically. Both are members of the transforming growth factor-beta superfamily, and their expression is confined to the oocytes. The major functions of this heterodimer are the promotion of folliculogenesis, modulation of granulosa cell responsiveness to FSH, and the increase in oocyte competence. In the present clinical context, exogenous FSH dose primarily reflects individualized treatment decisions based on patient characteristics such as age and ovarian reserve; therefore, it should not be interpreted as a direct biological regulator of zona remodeling. The BMP-15/GDF-9 heterodimer can be regarded, therefore, as a reliable marker of ovarian reserve and oocyte quality [39]. For technical reasons, we could only measure follicular fluid GDF-9 levels and assumed that its predictive value may be somewhat diminished compared with the BMP-15/GDF-9 heterodimer; however, follicular fluid GDF-9 alone proved to be useful in assessing the success or failure of embryo hatching. These observations are supported by reports demonstrating that GDF-9 is required for early ovarian folliculogenesis [40], and its mRNA levels in cumulus granulosa cells correlate with oocyte maturation, fertilization and early embryo development [41]. Furthermore, complete infertility occurs in GDF-9-deficient mice [40]. It is of interest that patients with BMP-15 mutations had a reduced ability to synergize BMP-15 with GDF-9, which might account for subsequent primary ovarian insufficiency (serum FSH > 40 IU/L, estradiol < 20 pg/mL) [42,43].

The effects of GDF-9 and other oocyte-derived members of the TGF-beta superfamily on oocyte growth and developmental potential are mediated by bidirectional feedback signaling between oocytes and cumulus cells. Specifically, factors secreted by the oocytes induce essential cumulus gene expression and facilitate cumulus expansion, while cumulus cells in turn supply the oocytes with the support necessary for healthy early embryo development [44]. Importantly, in the present study, the association of follicular fluid GDF-9 was confined to embryo hatching and did not extend to clinical pregnancy, indicating that GDF-9 primarily reflects early developmental competence rather than implantation success. However, given the observational nature of the present study and the limited cohort size, these mechanistic considerations should be regarded as supportive background rather than direct evidence of causality.

To explore further factors contributing to embryo hatching, attempts have been made to analyze the possible role of maturity-related changes in the composition of ZP. Namely, hyaluronic acid (HA) and syndecan-4 (Syn-4), the extracellular matrix-related components of the peri-oocyte, and the cumulus environment were measured in follicular fluid, assuming that their levels may reflect local extracellular matrix remodeling associated with oocyte maturation and zona pellucida dynamics. In our study, no associations could be detected between FF HA and Syn-4 with ZP characteristics and with embryo hatching/implantation; therefore, their significant contribution appears to be unlikely. In this context, it is to be considered that oocyte-derived members of the TGF-beta superfamily, in particular GDF-9, induce matrix expansion of the cumulus–oocyte complex (COC) with increased synthesis of HA and Syn-4 [45,46,47] that may be released into FF independent of ZP.

This study has several limitations. First, the sample size was relatively small (47 cycles, 64 embryos), which reduces statistical power and warrants caution when generalizing the results. Second, embryo-level observations were not fully independent because up to two embryos originated from the same patient. Although potential non-independence was explored in sensitivity analyses (e.g., one-embryo-per-patient re-fitting and GEE-based models), the small cluster size inherently limits statistical precision and should be considered when interpreting embryo-level associations. Third, follicular fluid biomarkers were limited to GDF-9, HA, and Syn4, and the biologically active BMP-15/GDF-9 heterodimer could not be measured, potentially underestimating the contribution of oocyte-derived regulatory pathways. Finally, this was a single-center observational study, and residual confounding cannot be excluded. The present study is based on a relatively small cohort (47 IVF cycles), and multivariable modeling, as well as discrimination metrics, should be interpreted as exploratory. Given the modest sample size and the inclusion of multiple exploratory analyses, an increased risk of a type I error and model overfitting cannot be excluded. External validation in larger, independent datasets is required to confirm reproducibility and generalizability. Despite these limitations, the overall consistency of the findings supports the biological relevance of the main observations. Δrel can be derived from routine time-lapse measurements without additional laboratory intervention. In practice, it may serve as an additional quantitative descriptor alongside established morphological and morphokinetic criteria. However, the present study does not establish incremental clinical utility, and prospective validation in larger cohorts is required before clinical implementation can be considered.

## 5. Conclusions

In conclusion, this prospective observational study demonstrates that dynamic zona pellucida thinning, quantified by the relative thinning ratio (Δrel), is strongly associated with embryo developmental competence. Δrel showed a consistent association with spontaneous blastocyst hatching and an independent association with clinical pregnancy at the patient level. Follicular fluid biomarkers, particularly GDF-9, were associated with early developmental events but did not independently predict clinical pregnancy. Together, these findings identify zona pellucida remodeling as a biologically plausible integrative marker linking oocyte-derived regulation and early embryo development, warranting further validation in larger, independent cohorts.

## Figures and Tables

**Table 1:** Comparison of clinical, biochemical, and zona pellucida (ZP) dynamic variables between hatching and non-hatching embryos and between pregnant and non-pregnant patients. Values are shown as mean ± SD. *p*-values from Welch’s *t*-test; ° *p* < 0.1, * *p* < 0.05.

Variable	Hatching (Mean ± SD)	No Hatching (Mean ± SD)	*p*-Value	Pregnant Mean ± SD	Not Pregnant Mean ± SD	*p*-Value
N	23	41	-	14	33	-
Relative zona pellucida thinning (Δrel, mean)	0.78 ± 0.16	0.28 ± 0.27	<0.001 *	0.61 ± 0.33	0.34 ± 0.30	0.0219 *
Zona pellucida thinning slope (mean)	−0.08 ± 0.02	−0.03 ± 0.04	<0.001 *	−0.08 ± 0.04	−0.03 ± 0.03	0.0025 *
Serum Syn4 (ng/mL)	8.82 ± 2.74	7.97 ± 2.32	0.3997	8.06 ± 2.45	8.26 ± 2.46	0.8169
Follicular fluid Syn4 (ng/mL)	7.40 ± 2.28	8.34 ± 3.39	0.2941	7.25 ± 2.17	8.46 ± 3.45	0.1574
Serum GDF-9 (pg/mL)	247.90 ± 112.04	189.53 ± 108.57	0.1738	232.93 ± 126.93	191.95 ± 103.02	0.3403
Follicular fluid GDF-9 (pg/mL)	2218.97 ± 889.98	1472.15 ± 542.97	0.0161 *	1869.36 ± 809.85	1575.20 ± 669.51	0.2448
Serum HA (ng/mL)	212.82 ± 102.33	145.43 ± 82.39	0.0825 °	157.81 ± 100.55	165.63 ± 89.28	0.8199
Follicular fluid HA (ng/mL)	494.00 ± 291.53	518.37 ± 231.81	0.7963	558.39 ± 313.20	492.53 ± 212.82	0.4809
BMI (kg/m^2^)	25.84 ± 7.22	26.22 ± 6.89	0.9006	25.39 ± 7.55	26.51 ± 6.63	0.6998
Maternal age (years)	31.42 ± 3.96	33.41 ± 3.50	0.1461	31.21 ± 4.56	33.67 ± 2.94	0.0839 °
Cumulative FSH dose (IU)	2188.33 ± 1356.26	2254.55 ± 665.24	0.8743	2045.36 ± 955.50	2333.59 ± 877.66	0.3557
Mature (MII) oocytes (*n*)	6.67 ± 5.03	3.89 ± 3.24	0.0988 °	5.23 ± 5.15	4.48 ± 3.41	0.6396
Mid-stimulation estradiol (E2, pg/mL)	2366.33 ± 1738.68	1209.52 ± 1401.79	0.0563 °	2082.43 ± 2386.83	1271.04 ± 866.07	0.2377

**Table 2:** Embryo-level logistic regression analysis of spontaneous hatching (*n* = 64 transferred blastocysts from 47 cycles). Odds ratios (ORs) with 95% confidence intervals (CIs) are shown for standardized predictors (per 1 SD increase). * *p* < 0.05.

Variable	OR	95% CI (Low)	95% CI (High)	*p*-Value
Intercept (constant)	0.27	0.10	0.72	0.009 *
Relative ZP thinning (Δrel, z-standardized)	5.94	2.23	15.81	<0.001 *
FF GDF-9 (z-standardized)	4.33	1.46	12.82	0.008 *
FF Syn4 (z-standardized)	0.75	0.30	1.88	0.53
FF HA (z-standardized)	1.15	0.57	2.31	0.70

**Table 3:** Variable contributions (%) to the first two principal components (PC1 and PC2) in embryo-level PCA of ZP dynamics (Δrel, slope) and clinical/follicular fluid predictors. Higher values indicate a stronger influence of the variable on the respective principal component.

Variable	PC1 Contribution (%)	PC2 Contribution (%)
Relative ZP thinning (Δrel)	30.8	1.5
ZP thinning slope	29.3	1.8
Maternal age (years)	11.0	1.9
BMI (kg/m^2^)	0.3	41.5
Cumulative FSH dose (IU)	19.0	8.5
FF GDF-9 (pg/mL)	8.4	2.8
FF Syn4 (ng/mL)	<0.001	41.6
FF HA (ng/mL)	1.2	0.4

**Table 4:** Generalized additive model (GAM) analysis of hatching probability in PCA space. Hatching status was modeled as a function of smooth terms for PC1 and PC2. Estimated degrees of freedom (EDF) indicate non-linearity (EDF > 1). ° *p* < 0.1; * *p* < 0.05.

Term	EDF	Ref.df	F-Value	*p*-Value
s(PC1)	2.917	3.647	20.656	<0.001 *
s(PC2)	1.000	1.000	4.037	0.0575 °

**Table 5:** Permutation ANOVA results from embryo-level constrained ordination. Global model fit is reported as the proportion of constrained inertia relative to total inertia (pseudo-R^2^). Marginal contributions represent R^2^ values from separate single-predictor models (999 permutations). ° *p* < 0.1; * *p* < 0.05.

Predictor	R^2^ (Marginal)	p (Permutation)
Global model fit (pseudo-R^2^)	0.308	0.022 *
Maternal age (years)	0.075	0.073 °
BMI (kg/m^2^)	<0.001	0.999
Cumulative FSH dose (IU)	0.213	0.003 *
FF GDF-9 (pg/mL)	0.090	0.036 *
FF HA (ng/mL)	0.018	0.420
FF Syn4 (ng/mL)	0.012	0.499

**Table 6:** Patient-level multivariable logistic regression analysis of clinical pregnancy (primary model: mean Δrel of transferred embryos). Odds ratios (ORs) with 95% confidence intervals (CIs) are shown for standardized predictors (per 1 SD increase). * *p* < 0.05.

Variable	OR	95% CI (Low)	95% CI (High)	*p*-Value
Intercept (constant)	0.59	0.17	2.07	0.410
Relative ZP thinning (Δrel, z-standardized)	3.65	1.39	9.62	0.009 *
FF GDF-9 (z-standardized)	0.90	0.40	2.03	0.801
FF Syn4 (z-standardized)	0.92	0.40	2.12	0.852
FF HA (z-standardized)	1.51	0.62	3.68	0.365
Assisted hatching (yes vs. no)	0.92	0.17	5.16	0.928

**Table 7:** Permutation ANOVA results from patient-level constrained ordination. Global model fit is reported as adjusted R^2^. Individual predictors are shown with permutation-based *p*-values (999 permutations). ° *p* < 0.1; * *p* < 0.05.

Predictor	p (Permutation)
Global model (adjusted R^2^ = 0.28)	0.005 *
Maternal age (years)	0.045 *
BMI (kg/m^2^)	0.937
Mid-stimulation estradiol (E2, pg/mL)	0.164
Cumulative FSH dose (IU)	0.004 *
Stimulation protocol (group)	0.709
Mature (MII) oocytes (*n*)	0.955
FF GDF-9 (pg/mL)	0.087 °
FF HA (ng/mL)	0.259
FF Syn4 (ng/mL)	0.970

## Data Availability

The data supporting the findings of this study are available from the corresponding author upon reasonable request.

## Supplementary Materials

The following supporting information can be downloaded at: https://www.mdpi.com/article/10.3390/jcm15052038/s1, Table S1: One-embryo-per-patient sensitivity analysis (outcome: spontaneous hatching); Table S2: Sensitivity analyses of clinical pregnancy models accounting for patient-level clustering; Table S3: Sensitivity analysis of embryo aggregation strategy in clinical pregnancy models (mean vs maximum Δrel); Table S4: Baseline characteristics stratified by assisted hatching (AH) status; Table S5: Additional analyses to enhance clinical interpretability of Δrel in clinical pregnancy prediction.

## Abbreviations

The following abbreviations are used in this manuscript:

ANOVA	Analysis of variance
AUC	Area under the receiver operating characteristic curve
BMI	Body mass index
BMP-15	Bone morphogenetic protein-15
CCA	Canonical correspondence analysis
CI	Confidence interval
EDF	Estimated degrees of freedom
ELISA	Enzyme-linked immunosorbent assay
E2	Estradiol
FF	Follicular fluid
FSH	Follicle-stimulating hormone
GAM	Generalized additive model
GDF-9	Growth differentiation factor-9
HA	Hyaluronic acid
Intercept (Constant)	Baseline odds at mean predictor levels in logistic regression
IU	International unit
MII	Metaphase II oocyte
OR	Odds ratio
PCA	Principal component analysis
PC1/PC2	Principal component 1/2
R^2^	proportion of variance explained Pseudo-R^2^ (CCA): proportion of constrained inertia/total inertia Adjusted R^2^ (CCA): bias-corrected explained variance (vegan::RsquareAdj)
Ref.df	Reference degrees of freedom (for GAM smooth terms)
ROC	Receiver operating characteristic
SD	Standard deviation
Slope	Linear thinning rate of the zona pellucida across time-lapse
Syn4	Syndecan-4
ZP	Zona pellucida
Δrel	Relative thinning ratio of the zona pellucida
