# Supplementary material for: Zona Pellucida Dynamics Integrate Biochemical and Clinical Indicators of Embryo Competence
Source: J Clin Med. 2026 Mar 7;15(5):2038. doi: 10.3390/jcm15052038 (PMC12985992; doi:10.3390/jcm15052038)
Supplement: Supplementary file 1 [file jcm-15-02038-s001.zip › jcm-4150787-supplementary.pdf]

Supplementary Table S1. One-embryo-per-patient sensitivity analysis (outcome: spontaneous hatching).

Variable	OR	95% CI (low)	95% CI (high)	p-value
Intercept (constant)	0.07	0.003	1.27	0.071°
Relative ZP thinning (Δrel, z-standardized)	21.41	1.49	308.64	0.024*
FF GDF-9 (z-standardized)	3.23	0.85	12.24	0.085°
FF Syn4 (z-standardized)	1.08	0.19	5.99	0.930
FF HA (z-standardized)	1.15	0.42	3.18	0.787

° $p < 0.1$; * $p < 0.05$

Continuous predictors were z-standardized prior to modeling (per 1 SD increase). Odds ratios (OR) with 95% confidence intervals (CI) are reported. The one-embryo-per-patient sensitivity analysis was based on random selection of a single embryo per patient. As expected given the reduced sample size and random selection, point estimates were less stable and confidence intervals widened accordingly.

Supplementary Table S2. Sensitivity analyses of clinical pregnancy models accounting for patient-level clustering.

(A) Generalized estimating equation (GEE) analysis with exchangeable correlation structure

Variable	OR	95% CI (low)	95% CI (high)	p-value
Intercept (constant)	0.58	0.21	1.63	0.304
Relative ZP thinning (Δrel, z-standardized)	3.72	1.45	9.51	0.006*
FF GDF-9 (z-standardized)	0.91	0.45	1.81	0.781
FF Syn4 (z-standardized)	0.92	0.45	1.89	0.825
FF HA (z-standardized)	1.51	0.61	3.72	0.369
Assisted hatching (yes vs no)	0.91	0.17	5.01	0.917

* $p < 0.05$

(B) One-embryo-per-patient sensitivity analysis

Variable	OR	95% CI (low)	95% CI (high)	p-value
Intercept (constant)	0.69	0.21	2.27	0.546

Variable	OR	95% CI (low)	95% CI (high)	p-value
Relative ZP thinning (Δrel, z-standardized)	2.36	1.00	5.59	0.050*
FF GDF-9 (z-standardized)	0.94	0.43	2.05	0.884
FF Syn4 (z-standardized)	0.86	0.37	1.98	0.717
FF HA (z-standardized)	1.15	0.49	2.69	0.751
Assisted hatching (yes vs no)	0.63	0.13	3.07	0.563

* $p < 0.05$

Continuous predictors were z-standardized prior to modeling (per 1 SD increase). Odds ratios (OR) with 95% confidence intervals (CI) are shown. The GEE model was fitted with an exchangeable correlation structure using patient identifier as the clustering variable. In the one-embryo-per-patient model, one embryo was randomly selected per patient to eliminate within-patient dependence.

Supplementary Table S3. Sensitivity analysis of embryo aggregation strategy in clinical pregnancy models (mean vs maximum Δrel).

(A) Mean Δrel (primary aggregation)

Variable	OR	95% CI (low)	95% CI (high)	p-value
Intercept (constant)	0.59	0.17	2.07	0.410
Mean Δrel (z-standardized)	3.65	1.39	9.62	0.009*
FF GDF-9 (z-standardized)	0.90	0.40	2.03	0.801
FF Syn4 (z-standardized)	0.92	0.40	2.12	0.852
FF HA (z-standardized)	1.51	0.62	3.68	0.365
Assisted hatching (yes vs no)	0.92	0.17	5.16	0.928

* $p < 0.05$

(B) Maximum Δrel (sensitivity aggregation)

Variable	OR	95% CI (low)	95% CI (high)	p-value
Intercept (constant)	0.68	0.24	1.95	0.464
Max Δrel (z-standardized)	2.27	1.03	4.98	0.041*
FF GDF-9 (z-standardized)	1.01	0.50	2.05	0.969

Variable	OR	95% CI (low)	95% CI (high)	p-value
FF Syn4 (z-standardized)	1.50	0.66	3.41	0.330
FF HA (z-standardized)	0.83	0.42	1.63	0.593
Assisted hatching (yes vs no)	0.69	0.13	3.64	0.658

* $p < 0.05$

Mean Δrel represents the arithmetic mean of transferred embryos in double embryo transfer cycles (primary model). Maximum Δrel represents the highest value among transferred embryos (sensitivity model). Continuous predictors were z-standardized.

Supplementary Table S4 Baseline characteristics stratified by assisted hatching (AH) status.

Variable	AH = No (n=30)	AH = Yes (n=17)	p-value
Maternal age (years)	31.54 ± 3.98	34.65 ± 2.37	0.003*
Previous IVF attempts	1.25 ± 0.44	2.53 ± 1.23	<0.001*
ZP thickness at 120h (μm)	9.62 ± 5.71	15.42 ± 6.70	0.008*
Clinical pregnancy (%)	11/30 (36.7%)	3/17 (17.6%)	0.30

* $p < 0.05$

AH was indication-driven (maternal age ≥37 years, ≥3 previous IVF failures, or ZP thickness >17 μm at 120 h). Continuous variables are presented as mean ± SD and compared using Welch's t-test. Pregnancy rates were compared using Fisher's exact test.

Supplementary Table S5. Additional analyses to enhance clinical interpretability of Δrel in clinical pregnancy prediction.

(A) Adjusted association between mean Δrel (per 0.1 increase) and clinical pregnancy

Predictor	OR	95% CI	p-value
Mean Δrel (per 0.1 increase)	1.45	1.10–1.91	0.009*

* $p < 0.05$

(B) Pregnancy probability by Δrel quartile

Quartile	n	Empirical pregnancy rate	Adjusted predicted probability
Q1	12	8.3%	8.7%
Q2	12	33.3%	18.9%
Q3	12	8.3%	36.1%
Q4	11	72.7%	57.8%
Linear trend p-value			0.011*

* $p < 0.05$

OR per 0.1 increment was derived from an unstandardized multivariable logistic regression model; quartiles were defined based on the distribution of mean Δrel across transferred embryos, and adjusted predicted probabilities were obtained from the same model.
